# Systematic Structure-Based Search for Ochratoxin-Degrading Enzymes in Proteomes from Filamentous Fungi

**DOI:** 10.3390/biom11071040

**Published:** 2021-07-16

**Authors:** Ana Lúcia Leitão, Francisco J. Enguita

**Affiliations:** 1 MEtRICs, Department of Sciences and Technology of Biomass, NOVA School of Science and Technology, FCT NOVA, Universidade NOVA de Lisboa, 2829-516 Caparica, Portugal; aldl@fct.unl.pt; 2 Instituto de Medicina Molecular João Lobo Antunes, Faculdade de Medicina, Universidade de Lisboa, Av. Prof. Egas Moniz, 1649-028 Lisboa, Portugal

**Keywords:** mycotoxin, ochratoxin A, ochratoxinase, biodegradation, structural biology

## Abstract

(1) Background: ochratoxins are mycotoxins produced by filamentous fungi with important implications in the food manufacturing industry due to their toxicity. Decontamination by specific ochratoxin-degrading enzymes has become an interesting alternative for the treatment of contaminated food commodities. (2) Methods: using a structure-based approach based on homology modeling, blind molecular docking of substrates and characterization of low-frequency protein motions, we performed a proteome mining in filamentous fungi to characterize new enzymes with potential ochratoxinase activity. (3) Results: the proteome mining results demonstrated the ubiquitous presence of fungal binuclear zinc-dependent amido-hydrolases with a high degree of structural homology to the already characterized ochratoxinase from *Aspergillus niger*. Ochratoxinase-like enzymes from ochratoxin-producing fungi showed more favorable substrate-binding pockets to accommodate ochratoxins A and B. (4) Conclusions: filamentous fungi are an interesting and rich source of hydrolases potentially capable of degrading ochratoxins, and could be used for the detoxification of diverse food commodities.

## 1. Introduction

Ochratoxins are an important group of mycotoxins produced by the secondary metabolism of some filamentous fungi. These toxic metabolites are ubiquitous contaminants of crops, seeds and processed food products, representing a serious threat to human health. Initially described as products of *Aspergillus ochraceus* [1], they were also characterized as metabolites from other fungal species belonging to the *Aspergillus*, *Penicillium* and *Byssochlamys* genera [2,3]. Chemically, the ochratoxin family of toxins is composed of polyketide compounds with a basic nucleus harboring an isocoumarin molecule connected with an L-phenylalanine by an amide bond. Ochratoxins occur in three natural isoforms (Ochratoxins A, B and C), whereby Ochratoxin A (OTA) is the most abundant and toxic. OTA contains a covalently bound chlorine atom in the isocoumarin ring, which is absent in Ochratoxin B (OTB). Ochratoxin C (OTC) is an acetyl derivative that in biological systems is rapidly hydrolyzed to generate OTA.

The presence of a phenylalanine residue in ochratoxins initially pointed to the possibility that their toxic effects were mediated by an inhibition of the metabolism of this amino acid. Early experimental evidence showed that OTA was able to interact with the phenyl-alanyl-tRNA synthetase, acting as an inhibitor of the protein synthesis in bacterial cells [4]. Further studies demonstrated additional biological activities associated with ochratoxin toxicity. OTA has been characterized as a mutagenic agent by direct interaction with nucleic acids and induction of DNA damage [5], showing specific toxicity to the kidneys, liver and immune system [6,7]. The OTA-induced toxicity is especially severe in humans due to its increased bioavailability when compared with other mammals [8,9]. International and national legislations limit the presence of ochratoxins in food and human exposure to the toxins. The European Food Safety Authority (EFSA) performed a combined study determining OTA concentrations in relevant foods (cereals, fruits, beverages and meat) and their toxicity on animal models to establish a tolerable weekly intake (TWI) of 120 ng/kg body weight [10].

The absence of ochratoxins in crops and manufactured food products is heavily dependent on avoiding the fungal colonization of raw material. However, due to logistic and processing limitations of food components, fungal contamination is often very difficult to abolish. In consequence, several methods to decontaminate and detoxifying food commodities have been described in the literature [11]. Physical methods for OTA decontamination are not very efficient due to OTA’s intrinsic stability. Heating up to 250 °C [12,13], intermittent ultrasonic treatment in an aqueous medium supplemented with alkali [14], gamma irradiation [15] and ultrafiltration [16] have been used to reduce the levels of OTA in different food commodities. Other detoxifying protocols include the adsorption over minerals or modified clays [17] and also chemical methods such as the treatment with reducing and alkylating agents [18,19]. In general, physico-chemical and chemical protocols for ochratoxin detoxification have the major issue of induced alterations in the organoleptic properties of food commodities. In consequence, biological detoxification methods based on the use of enzymes are a very interesting alternative.

The different bioavailability of ochratoxins in mammals prompted the possibility of the existence of microorganisms able to degrade these mycotoxins and established as members of the internal microflora [20]. Microflora extracted from the gastrointestinal tract of ruminants were able to degrade different mycotoxins, including OTA [21]. The presence of this rumen microflora was described as essential to understanding the inherent resistance of herbivores to OTA [22]. Environmental bacterial and fungal isolates have been also described as able to degrade different ochratoxins [23,24,25].

**Figure 1:**
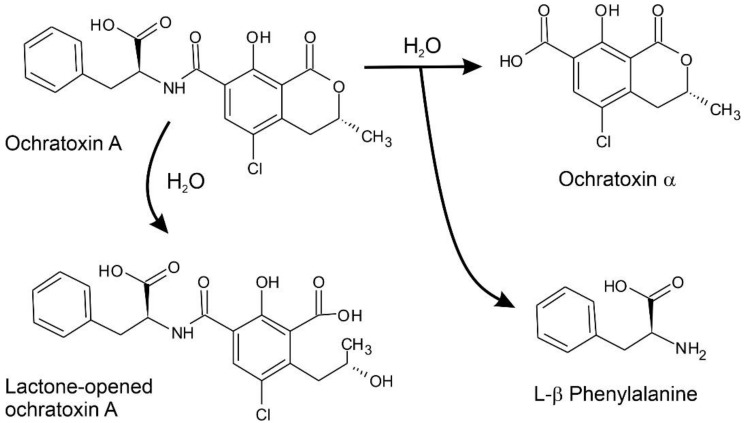
Enzymatic hydrolysis of OTA. Ochratoxinases with amido-hydrolase activity are able to break the amide bond in OTA, releasing two non-toxic products: ochratoxin α and L-β-Phenylalanine. Lactone-hydrolases can also produce harmless metabolites by opening the OTA lactone ring, but they are less efficient than ochratoxinases.

The enzymatic detoxification of ochratoxin could be achieved by two different hydrolysis reactions: hydrolysis of the amide bond to generate the harmless metabolite ochratoxin α and L-phenylalanine by the involvement of an amido-hydrolase, or the hydrolysis of the lactone ring by an ochratoxin-lactonase (Figure 1) [26]. Early evidences demonstrated that proteolytic enzymes could degrade ochratoxins, generating non-toxic products [27]. However, in the last decade, an increased effort for the discovery and characterization of more specific enzymes with ochratoxinase activity has been made [28,29]. At this moment in time, the best-characterized enzyme catalyzing ochratoxin hydrolysis is the ochratoxinase from the ochratoxin producer *Aspergillus niger*, a binuclear Zn-dependent amido-hydrolase with striking sequence homology with other amido-hydrolases and peptidases. The enzyme structure has been characterized by X-ray crystallography studies, which allowed to determine a detailed map of the catalytic residues and to propose a reaction mechanism. Ochratoxinase-mediated catalysis joins the unique structure of the substrate-binding pocket to the production of a favored reaction intermediate shared by both Zn^2+^, which enhances its catalytic yield in comparison with other proteolytic enzymes [30]. Considering the metabolic diversity of filamentous fungi, the objective of the present work is to characterize new enzymes with potential ochratoxinase activity selected from fungal proteomes. To circumvent the intrinsic conservation of amido-hydrolases across proteomes, we used a specific structure-based approach for the mining and characterization of fungal amido-hydrolases that could potentially be useful for bioremediation applications. Our work is complemented by a complete characterization of substrate-binding pockets and protein dynamics using the tridimensional structure of *A. niger* ochratoxinase as a reference.

## 2. Materials and Methods

### 2.1. Fungal Proteome Interrogation

Sequence homologs of *A. niger* ochratoxinase (UniprotKB reference: A2R2V4) in fungal proteomes were selected by database searching using the BLASTP algorithm [31]. The hit list was filtered by *p*-values and query coverage, considering only the non-redundant protein homologs with *p*-value < 10^−15^ and query coverage > 75%. The final list of selected fungal putative ochratoxinases comprised 83 elements.

### 2.2. Homology Modeling, Model Refinement and Substrate Docking

The atomic structure of the putative selected ochratoxinases was predicted by homology modeling and threading using the Phyre2 algorithm [32]. The structural models of each protein were built applying the automated mode of the algorithm, and filtered to ensure a confidence level of 100%. The atomic coordinates of the Phyre2 homology models were refined by a two-step atomic-level energy minimization using the ModRefiner algorithm [33]. The refined atomic models were further visually inspected for the localization of the enzyme active center and the catalytic residues [34].

Atomic models of ochratoxins A, B and C were manually built with Maestro software v.12.3 (Schrödinger, Manheinn, Germany). Hydrogen atoms were automatically added following the riding model and the structures were optimized by 100 cycles of energy minimization. Molecular docking of ochratoxins A and B on the surface of the protein models was performed by applying a blind method with a previous cavity detection and flexible docking using CB-Dock and Autodock Vina [35]. This method allows an unbiased characterization of substrate-binding patterns that is not dependent on the previous knowledge of the structure–function relationships of the studied proteins. The list of docking solutions was ranked by their internal scores and Gibb’s function.

### 2.3. Molecular Dynamics Simulations

The structure and stability of the ochratoxinase enzyme from *A. niger* in apo-form and complexed with OTA was studied by molecular dynamics simulations with the GROMACS suite v. 5.1.2. using a full-atom model strategy and the CHARMM27 force field parameters [36]. The topology files and input scripts for the simulations were generated by the CHARMM-GUI suite [37]. Briefly, the apo-enzyme and OTA complex were placed in the center of cubic boxes, solvated with TIP3P water molecules and neutralized by the addition of potassium and chloride ions to a concentration of 0.150 mM. The coordinates of the system were then minimized by 5000 cycles of the steepest descent method. After system minimization, an NVT ensemble (V-rescale thermostat with constant number of particles, system volume and temperature) followed by an NPT ensemble (Parrinello–Rahman barostat with constant number of particles, pressure and temperature) was performed for 1 ns at 1 atm and 300 K to ensure the equilibration of the system prior the simulations. The production molecular dynamics simulations were further continued at 300 K for 10 ns using the LINCS linear constraint solver and a simulation step of 2 fs. The generated trajectories were analyzed by the internal GROMACS analysis tools and complemented by the use of the MD-TASK suite [38].

### 2.4. Calculation of Substrate-Binding Free Energy from Molecular Dynamics Simulations

The molecular dynamics trajectories obtained in 2.3 were used to characterize and model the interactions between OTA and the *A. niger* ochratoxinase. Among all the available protocols for the characterization of protein–ligand interactions, we selected the molecular mechanics Poisson–Boltzmann surface area method (MM-PBSA), due to its efficiency and reliability in small- to medium-sized molecular systems [39]. Following the MM-PBSA protocol, the energy of binding of an enzyme–substrate complex is defined as the difference between the free energy of the enzyme–substrate complex and the corresponding free energies of the isolated protein and substrate in an aqueous environment [40]. Separate free energies of each component are calculated following Gibb’s equation, where the enthalpy term is represented by the average molecular mechanics (MM) potential energy in a vacuum, as previously described [39,40].

The molecular dynamics trajectory files from *A. niger* ochratoxinase complexed with OTA were analyzed by the g_mmpbsa package [41] to determine the substrate-binding energies of the complex along the simulation. The energy decomposition and determination of the relative contribution of each amino acid residue to the stabilization of the enzyme–substrate complex was performed by the use of the ‘MmPbSaDecom.py’ Python script included in the g_mmpbsa package, considering the contribution of the molecular mechanic’s energy, the polar and apolar solvation energies and following a bootstrap analysis strategy with 2000 steps [41].

### 2.5. Structure Comparison

The refined atomic models of putative ochratoxinase enzymes were superposed in 3D space by the Caretta software, a recent protein-structure-alignment algorithm that combines dynamic time warping together with progressive pairwise alignment to superpose multiple protein structures in a very efficient manner [42]. The same algorithm allowed to build a sequence alignment of all the models based on their structural superposition. This protocol allows maximizing the characterization of conserved amino acids positions across all the structures and extracting the corresponding functional residues. The structure-based sequence alignment was further used to construct a phylogenetic tree that was represented and analyzed by the iTOL software v5.0 [43].

The degree of evolutionary conservation of the amino acids across ochratoxinase structure models was mapped over the coordinates of *A. niger* ochratoxinase (PDB code: 4C5Y) by Consurf 2016 software [44].

### 2.6. Structure Analysis

The electrostatic surface properties of protein models were determined by the Adaptive Poisson–Boltzmann Solver (APBS) [45] and represented on the solvent-accessible molecular surfaces with Pymol [46] and CCP4MG [47].

Large-scale protein movements were studied by low-frequency normal mode analysis using a C-alpha force field [48]. This strategy employs a coarse-grained molecular modeling approach where the whole molecular mass of every amino acid is assigned to its alpha-carbon. Deformation energies and normalized atomic displacement parameters were calculated and interpreted by using the WEBnm@ v. 2.0 software [49].

Additional graphical representations of atomic coordinates and their properties were performed with the Protein Imager software [50].

## 3. Results

### 3.1. Catalytic Center of Ochratoxinase from A. niger

The already described catalytic center of the ochratoxinase from *A. niger* (PDB code: 4C5Y) was used as a structural reference for the characterization of putative ochratoxinase-hydrolyzing enzymes in other fungi [30]. *A. niger* ochratoxinase is a binuclear Zn-dependent hydrolase, containing a conserved arrangement of catalytic residues also present in other hydrolases and esterases [51]. The first Zn^2+^ ion is coordinated by two histidine (His111 and His113) and one aspartate (Asp378) residues, whereas the second Zn^2+^ has partial coordination ensured by His287 and His307. The remaining coordination position is used by the enzyme to stabilize the chemical intermediate prior to the hydrolysis of the substrate. Both Zn^2+^ ions are immobilized by a covalently modified acetyl-Lysine residue (Ac-Lys246), which is essential for the proper functionality of the enzyme.

**Figure 2:**
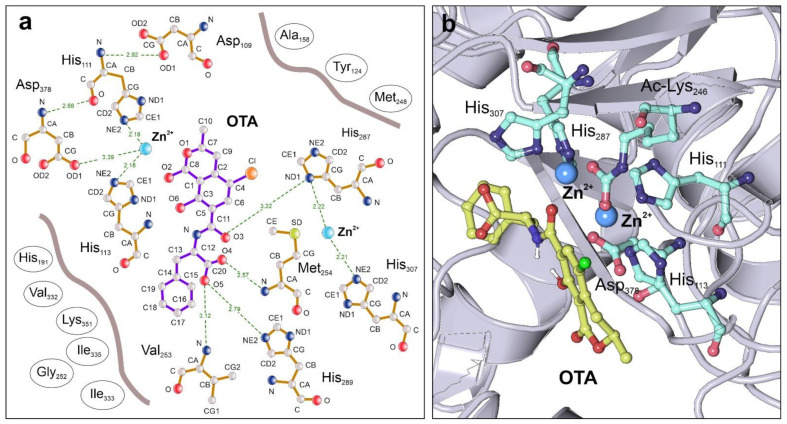
Catalytic residues and molecular interactions of *A*. *niger* ochratoxinase (PDB code: 4C5Y) determined by blind molecular docking. (**a**) Two-dimensional Ligplot diagram [52] showing the interactions established between the substrate OTA and the residues in the catalytic pocket, as determined by cavity detection and blind docking with the CB-Dock software [35]. Hydrogen bonds are depicted by dotted lines showing the distances between the interacting atoms represented in Angstroms. The amino acids configuring the substrate-binding pocket and establishing other molecular interactions with OTA are represented by circles.; (**b**) Tridimensional view of the enzyme catalytic pocket in complex with the OTA substrate, showing the presence of the modified residue Acetyl-Lys246 that is essential for the stability of the binuclear Zinc center, and the remaining metal-coordinating amino acids. This representation has been prepared with the Protein Imager software [50].

To characterize the complete substrate-binding pocket of ochratoxinase, we performed a blind docking experiment using OTA and OTB as ligands (Figure 2). Our results showed a preferred docking solution where OTA and OTB substrates are bound to a deep protein surface pocket located near the binuclear Zn center. Calculated free energies of the enzyme–substrate complex are more favorable for the OTA binding to the enzyme catalytic center. The substrate-binding pocket is composed of a combination of hydrophobic and polar residues that establish interactions with the coumarin ring of ochratoxins. There are two partially conserved residues in other amido-hydrolases, His289 and Met254, establishing hydrogen–bond interactions with the carboxyl group of phenylalanine chain and the carbonyl group in the coumarin ring, respectively. All these interactions could contribute to the stabilization of the substrate in the catalytic pocket. We also postulate the involvement of Lys351 in substrate-binding by establishing cation–pi interactions with the aromatic rings in OTA and OTB. Our data also suggested that an additional OTA-interacting methionine residue, Met248, located at the edge of the substrate-binding pocket, could be important for the proper configuration of the catalytic pocket of the enzyme (Figure 2).

### 3.2. Characterization of OTA-Binding Pocket by Molecular Dynamics

**Figure 3:**
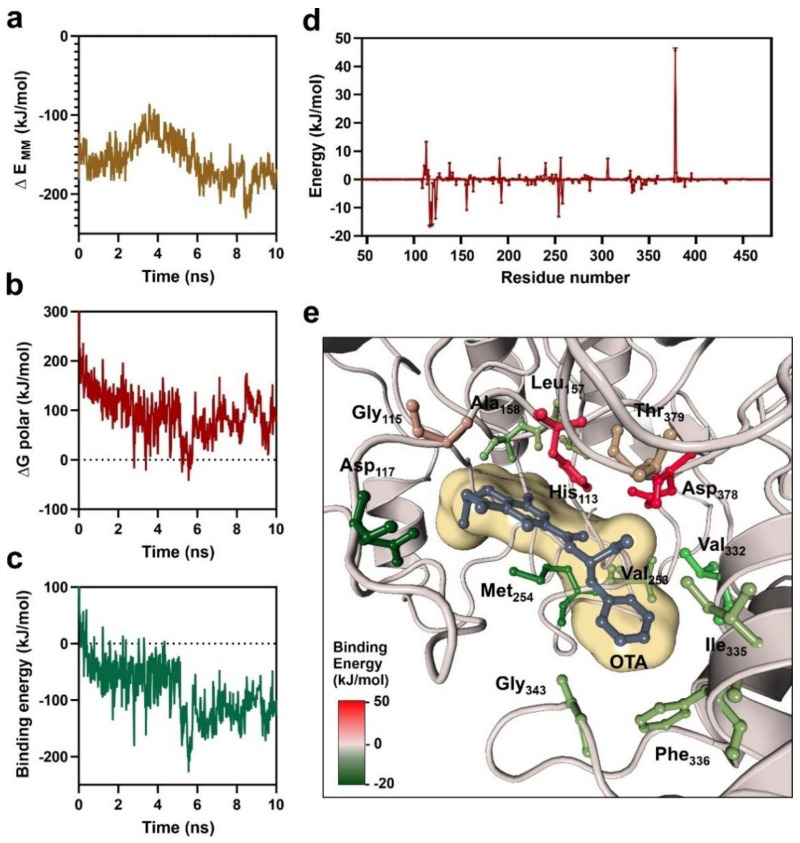
Analysis of the *A. niger* ochratoxinase enzyme–substrate complex by the molecular mechanics Poisson–Boltzmann surface area method (MM-PBSA). (**a**) Time course of the average molecular mechanics potential in vaccum during the molecular dynamics simulation; (**b**) time course of the average polar free energy of solvation during the simulation; (**c**) time course of the average OTA-binding free energy during the simulation; (**d**) contribution of each individual residue to the binding energy of the OTA–enzyme complex showing the average values for each amino acid in the *A. niger* ochratoxinase; (**e**) detailed view of the substrate-binding pocket, depicting the amino acids located at a distance <4 Å to the OTA substrate which are colored according to their relative contribution to the binding energy of the substrate.

Molecular docking protocols combined with molecular dynamics simulations have been extensively used in computer-aided drug design due to their flexibility and easy implementation; however, their use in the characterization of enzyme–substrate complexes is quantitatively less extended [53]. To study the dynamics of the OTA substrate-binding to the ochratoxinase from *A. niger*, we applied a classical molecular dynamics simulation in a controlled aqueous environment combined with molecular mechanics Poisson–Boltzmann surface area analysis (MM-PBSA). The obtained results are depicted in the Supplementary Materials Figures S2 and S3 and in Figure 3.

Despite the short simulation time of 10 ns designed for the characterization of the substrate-binding dynamics, the comparison of the simulation trajectories from the apo-enzyme to the OTA–enzyme complex showed a striking differential pattern in which respect to the gyration radius and relative abundance of secondary structure elements. The OTA–enzyme complex behaves as a more compact system when compared to the apo-enzyme, as demonstrated by the evolution of the average gyration radius and all its components (Supplementary Materials Figure S3a,b). Substrate-binding to the *A. niger* ochratoxinase also induces a folding transition of the enzyme, represented by an increase in the average number of residues located in coiled regions and a decrease of the residues in beta-sheets and alpha-helices along the molecular dynamics simulation (Supplementary Materials Figure S3c,d).

The binding energy of the OTA substrate to the ochratoxinase enzyme was determined by MM-PBSA analysis of the molecular dynamics trajectories. MM-PBSA method is an efficient and reasonable approximation that can be used as a convenient alternative to quantum mechanics calculations which are more complex and time-consuming [53,54]. Key points that increase the efficiency of MM-PBSA are the assumption of the existence of a continuous solvent distribution within the studied system and the approximation of the entropy and enthalpy values for each component of the system (enzyme and substrate) [39]. Applying the MM-PBSA analysis to the simulation trajectories of the OTA–enzyme complex, we determined an average substrate-binding energy of −84.945 ± 2.258 kJ/mol, which could be decomposed into contributions from van der Waal interactions (−196.626 ± 0.517 kJ/mol), electrostatic energy (39.646 ± 1.181 kJ/mol), polar solvation energy (93.662 ± 2.085 kJ/mol) and solvent-accessible surface energy (−21.635 ± 0.041 kJ/mol). The time-course evolution of the binding energy along the molecular dynamics simulation is depicted in Figure 3c.

The binding energies for the enzyme–substrate system were decomposed to calculate the relative contribution of each amino acid residue to the substrate-binding energy. The relative contribution to the system energy indicates if a given residue has a positive or negative impact over the free-binding energy of the enzyme–substrate complex, and it is not necessary related with the direct interaction of the residue with the substrate [41,54]. The results of this systematic decomposition are available as Supplementary Materials Table S3 and represented in Figure 3d,e, respectively. Among the residues located at an average distance < 4 Å from the OTA substrate during the simulation, the catalytic Asp378 and the His113 involved in the coordination of one Zn atom have a negative contribution to the free binding energy of the enzyme–substrate complex. However, the residues located in the boundaries of the catalytical pocket (Asp117, Leu157, Ala158, Met254, Val332, Ile335, Phe336 and Gly343) have a positive contribution to the free energy of the system. Among them, Ala158, Met254, Val332 and Ile335 were also characterized as OTA-interacting residues as described in our blind docking experiments.

### 3.3. Fungal Proteome Mining for the Presence of Putative Ochratoxinases

The fungal proteomes deposited at the NCBI databases were scanned for ochratoxinase homologs by using the sequence of *A. niger* enzyme (Uniprot ID A2R2V4) and the BLASTp searching algorithm [55]. Since the amido-hydrolase family of enzymes is widespread across all the living cells, we imposed strong restrictions for finding ochratoxinase sequence homologs (*p*-value < 10^−15^ and query coverage > 75%). After the elimination of redundant entries, we built a dataset composed of 83 protein sequences. All the selected protein sequences were used as seeds for homology modeling with the automated algorithm implemented in Phyre2 [32]. Filtering the homology modeling solutions by using a 100% degree of confidence, the hit templates used for the modeling were the *A. niger* ochratoxinase in 77% of the sequences (PDB codes: 4C65 and 4C5Y), and two amido-hydrolases of unknown origin characterized from a metagenomic project in 23% of the cases (PDB codes: 3MKV and 3FEQ). The detailed results of the Phyre2 homology modeling process are included in Supplementary Materials (Table S1), together with the individual atomic models in PDB coordinate format.

**Figure 4:**
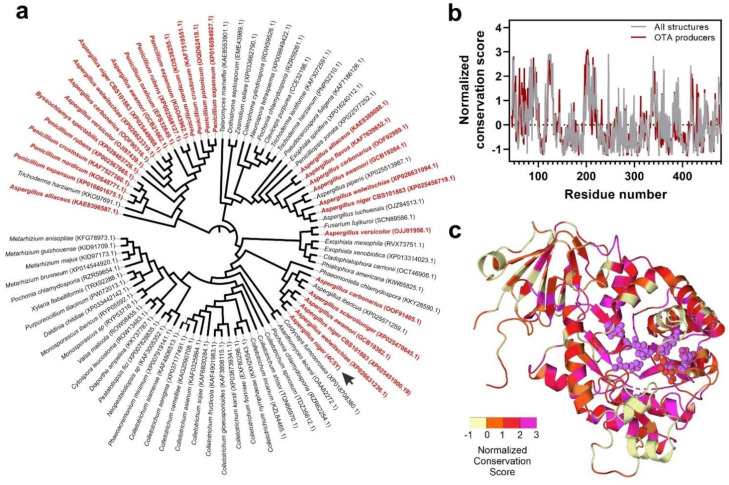
Structure comparison of the selected set of fungal ochratoxinase-like atomic models. (**a**) Unrooted phylogenetic tree constructed by the iTOL software [43] and based on the structural superposition of all the atomic models using the Caretta algorithm [42]. The enzyme models are identified by the source microorganism and the ID code from the Uniprot database. Names in red color are enzymes belonging to already characterized ochratoxin producers, whereas the remaining sequences were extracted from proteomes of other fungal strains. The position of *A. niger* ochratoxin is labeled with an arrow. (**b**) Normalized conservation score of the whole family of selected proteins calculated by the ConSurf 2016 server [44] using the previous structural superposition and plotted over the sequence of *A. niger* ochratoxinase. The ConSurf conservation score calculated for the ochratoxinase-like enzymes extracted from ochratoxin-producing fungal proteomes (red line) is compared with the same score calculated for all the cohort of proteins (grey line). (**c**) Spatial representation of the normalized conservation scores of the ochratoxinase-like enzymes from ochratoxin-producing fungi over the atomic model of *A. niger* ochratoxinase (PDB code: 4C5Y). The residues involved in the catalytic activity of the protein are highlighted and represented by atomic spheres.

The structural superposition of the refined atomic models followed by a phylogenetic analysis allowed us to determine that the ochratoxinase-like enzymes belonging to ochratoxin-producing fungi can be clustered into three groups (Figure 4a). Two groups are integrated by enzymes belonging to species of the *Aspergillus* genus, whereas the third one also includes species of *Penicillium* and *Byssochlamys* genera. The structural conservation of residues in specific spatial positions was further analyzed by considering the results of the structure-based sequence alignment of the selected enzymes and using the ConSurf algorithm [44]. The ConSurf method calculates a conservation score in a sequence alignment that can be represented along a reference sequence or structure. This score can be interpreted in evolutionary terms but also is extremely useful for inferring functional relationships within a family of proteins. The results of this analysis are depicted in (Figure 4b,c). Using *A. niger* ochratoxinase as a working reference, the normalized conservation score plot along its sequence showed the presence of different conserved subdomains in the analyzed enzymes, which comprise the catalytic site and the protein segment that connects both enzyme domains (Figure 4b). Interestingly, the conservation profile plot is different when we analyzed the group of ochratoxinase-like enzymes selected from ochratoxin-producing fungi. These enzymes have a conserved region in the N-terminal segment, comprising residues from 50–80, which is less conserved in the remaining dataset. Considering the structure of *A. niger* ochratoxinase, the conserved region corresponds to the amino acids located in the segment that stabilizes the connection between both protein domains, being located close to the catalytic pocket. Careful observation of the ConSurf normalized score in the ochratoxinase-like enzymes selected from ochratoxinase producers and plotted over *A. niger* ochratoxinase supported the conclusion that the catalytic center comprising the Zn-coordinating residues and the catalytic pocket is highly conserved (Figure 4c). However, the external limits of the substrate-binding cavity are more variable across the enzymes, suggesting the possible involvement of this region in the substrate specificity.

### 3.4. Substrate Docking and Characterization of Substrate-Binding Cavities

**Figure 5:**
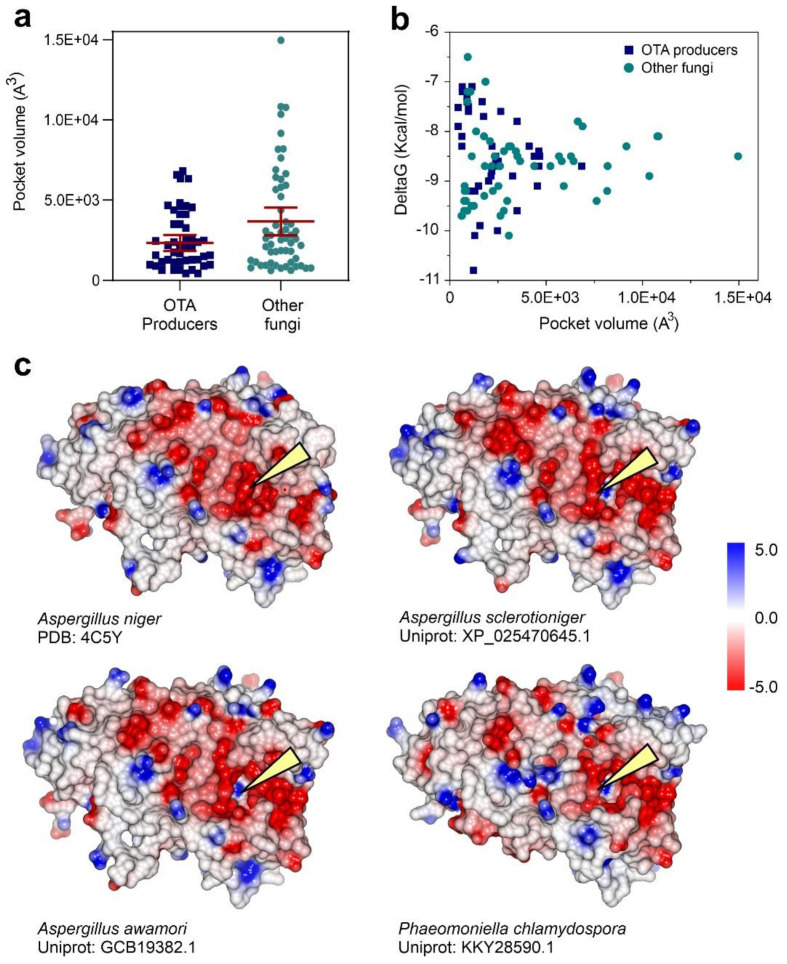
Characteristics of the substrate-binding pockets in the dataset of fungal ochratoxinase-like enzyme models. (**a**) Volume of the substrate-binding pocket determined by the CB-Dock algorithm [35], distinguishing between the enzymes encoded by ochratoxin producers and other fungi. (**b**) Results of the blind docking and pocket search analysis by CB-Dock with OTA as ligand. The plot represents the relationships between the substrate-binding pocket volume in each enzyme model and the calculated Gibbs’ function value for the best docking solution of the ligand. Following the same criteria, the enzyme models are classified according to their original fungal strain. (**c**) Surface potential representation of selected structural models of the analyzed dataset. The reference structure of *A. niger* ochratoxinase, together with the enzymes from *A. sclerotioniger* and *A. awamori*, are examples of ochratoxinase enzymes from fungal strains characterized as toxin producers, whereas the enzyme from *P. chlamydospora* is an example of other fungal species not described as ochratoxin producers. The location of the substrate-binding pocket entrance is depicted by a yellow triangle. The electrostatic potential scale is depicted as a gradient color bar.

The ability of an amido-hydrolase to hydrolyze ochratoxins would be highly dependent on the configuration of the substrate-binding pocket, which could influence its accessibility and determine the substrate-binding affinity. Our systematic search for ochratoxinase-like enzymes in fungal proteomes was complemented by a blind docking analysis using OTA and OTB as enzyme substrates and the refined homology models of the fungal enzymes as receptors. The best docking solutions involving the interaction of OTA or OTB with the substrate-binding pocket were selected and ranked by their respective Gibbs’ function value. The complete results of the analysis are presented in Supplementary Materials Table S2 and summarized in Figure 5.

The volume of the substrate-binding pockets in the enzymes from ochratoxin producers had an average value of 2342.9 ± 1516.5 Å^3^, whereas the proteins in the non-ochratoxin producers showed catalytic pockets with an average volume of 3742.9 ± 3264.4 Å^3^ (Figure 5a). Interestingly, the free energy of the protein–ligand complexes determined by the CB-Dock algorithm also showed a differential pattern in both groups of enzymes. Catalytic pockets from OTA-producing fungi have more favorable binding energies for both substrates OTA and OTB when compared with the enzymes from other fungi (Figure 5b). The increased stability of enzyme–substrate complexes together with the presence of narrower binding pockets suggested an augmented specificity of these enzymes for their substrates.

The analysis of the amino acids located at the catalytic cavity of the enzymes demonstrated the presence of conserved residues in the group of ochratoxinase-like enzymes from OTA-producing fungi (Supplementary Materials Figure S4). Besides the Zn-coordinating amino acids, five other residues are conserved in the ochratoxinase-like enzymes from OTA producers, all of them involved in molecular interactions with the substrate. Considering the structure of *A. niger* ochratoxinase as a reference, the conserved residues are Tyr124, His191, Met248, His289 and Lys351 (Figure 2a and Supplementary Materials Figure S4). His191 and Lys351 are present in all the analyzed enzymes and, following the results of our docking experiments; they are apparently involved in the stabilization of the enzyme–substrate complex by binding to the ochratoxin ring by hydrogen bonds (His191) or by the establishment of cation–pi interactions (Lys351). Met248, located close to the Zn-coordinating AcLys246, is conserved in approximately two-thirds of the analyzed enzymes and involved in direct interactions with the substrate and stabilization of the main entrance of the catalytic pocket. Position 289 is either occupied by histidine or a tyrosine residue, being important for the stabilization of the substrate close to the catalytic ions. Finally, Tyr124, that in some of the enzymes is substituted by arginine or a proline residue, establishes direct hydrophobic interactions with the coumarinic ring of ochratoxin. The conservation of these five residues in other ochratoxinase-like enzymes is less evident, as concluded from the structural superposition of the atomic models.

The catalytic pocket containing the di-nuclear Zn center is located in a deep protein cavity, accessible to the exterior by a narrow molecular channel. The entrance of the substrate channel is located in a protein face characterized by a clear pattern of negative electrostatic potential (Figure 5c). The analysis and comparison of the electrostatic surface potentials and distances obtained by calculation of the Poisson–Boltzmann equation across all the ochratoxinase enzymes revealed the differences between the group of enzymes from ochratoxin producers and non-producers in a pattern that resembles the already-observed differences in the atomic structure (Supplementary Materials Figure S5).

### 3.5. Characterization of Low-Frequency Domain Motions

**Figure 6:**
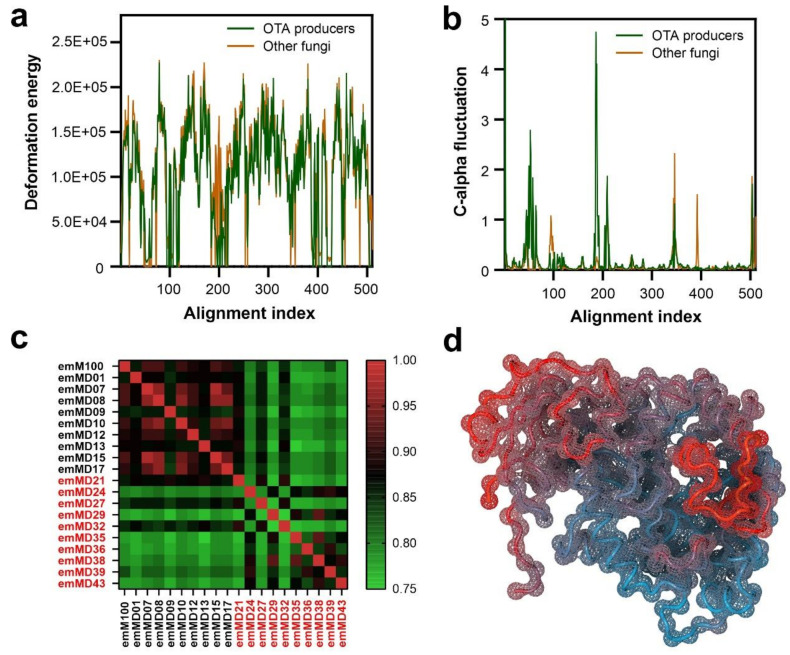
Normal mode analysis of a subset of ochratoxin-like enzymes applying the webNM@ platform [49]. (**a**) Average of the deformation energy calculated along the protein sequences in the ochratoxin-like enzymes distinguishing ochratoxin producers and non-producers. (**b**) C-alpha space fluctuations along the normal mode simulations, showing the average movements in the ochratoxin-like models. (**c**) Bhattacharya Coefficient (BC) scoring matrix of the selected subset of ochratoxinase-like enzymes. The enzymes models are identified by their internal codes (Supplementary Materials Table S1). Enzyme names belonging to ochratoxin-producing fungi are colored in red. (**d**) C-alpha space fluctuations calculated by normal mode analysis and represented over the structure of the *A. niger* ochratoxinase (PDB code: 4C5Y). The color of the protein trace represents the relative fluctuations of the C-alpha along the simulation following a gradient (blue to red, from more static to more fluctuating regions of the protein).

The characterization of protein dynamics from structural models intends to describe the presence of mobile or rigid protein regions and their functional relationships. Normal mode analysis is an efficient alternative approach for the study of protein domain motions when compared with classical molecular dynamics methods, where the biological systems are defined at the atomic level [48]. Normal mode uses a method based on coarse-grained models that allow determining low-frequency motions in proteins and other biomolecules [56]. We applied a normal mode analysis for characterization of the domain motions across the cohort of ochratoxinase-like atomic models with a special focus on the substrate-binding domain by applying the WebNM@ algorithm [49]. The study includes individual analysis for each of the atomic models together with a comparative analysis comprising 10 members of each of the described group of enzymes in order to calculate the deformation energies and C-alpha fluctuations associated with each amino acid residue (Figure 6).

In the comparative deformation energies plot (Figure 6a), we observe that all the ochratoxinase-like structures behave following a common trend represented by the existence of two clear regions of higher deformation energies separated by a flexible segment (alignment index from 190–210). Interestingly, this valley of lower deformation energies is more pronounced in the group of enzymes belonging to ochratoxin-producing fungi. The amino and carboxy-terminal regions of all the proteins are also characterized by lower deformation energies. Moreover, the flexibility of specific protein regions can also be stated by the analysis of the C-alpha fluctuations (Figure 6b). Amido-hydrolases from ochratoxin producers showed two regions with increased main-chain fluctuations when compared with the remaining group of enzymes from non-producers. These regions correspond to the N-terminal domain (alignment index from 40 to 60), and to a central segment (alignment index from 190–210). The central mobile flexible segment was previously mentioned and also characterized by a decreased deformation energy. Upon a careful structural models inspection, we determined that this region is composed of two alpha-helices located in the limits of the substrate-binding pocket that can be clearly observed when the low-frequency movements are represented on the C-alpha trace of the *A*. *niger* ochratoxinase structure (Figure 6d). Ochratoxinase-like enzymes from the ochratoxin producers showed increased flexibility and predicted low-frequency movements in this region, which suggest an increased capacity for the accommodation of substrates.

The webNM@ algorithm also allowed to calculate a comparison matrix based on the Bhattacharya Coefficient (BC). The matrix represented in Figure 6c provides a measure of the similarity between the intrinsic motions of the proteins but does not inform which parts of the structures are responsible for such differences. The group of enzyme models from fungi not characterized as ochratoxin producers showed a very homogeneous pattern of intrinsic motions characterized by higher values of BC coefficient. The homogeneity of this group of proteins is in clear contrast to the enzymes from the ochratoxin producers (Figure 6c).

## 4. Discussion

Bioremediation of ochratoxins from food commodities by the use of enzymes is an interesting alternative to the chemical or physical treatments that can alter their nutritional composition and organoleptic properties [11]. Among all the biochemical reactions to degrade ochratoxins, the enzymatic hydrolysis of the amide bond that links the coumarin ring and the phenylalanine residue offers clear advantages since it can occur at low temperatures and it releases two harmless degradation products, ochratoxin α and L-β-phenylalanine [57]. Despite its intrinsic applicability for ochratoxin detoxification, the enzymatic hydrolysis of its amide bond could be potentially catalyzed by a myriad of different enzyme families. Proteases and amido-hydrolases from different origins have been demonstrated to hydrolyze ochratoxins with different yields [27,28,29]. The most efficient enzyme already known is an ochratoxinase from *A. niger*, a Zn-dependent amido-hydrolase that is able to degrade OTA and OTB [30]. However, the portfolio of potentially applicable detoxifying enzymes is still limited, and consequently, there is an unmet need for the discovery and application of new ochratoxin-degrading enzymes that could be used in food detoxification. As secondary metabolites producers, filamentous fungi are an interesting source of enzymatic diversity and consequently an important niche for the discovery of new ochratoxin-degrading enzymes [23,58].

High-throughput screening and data mining strategies are ideal for the discovery of new ochratoxin-degrading enzymes. Due to the intrinsic conservation of the protease and amido-hydrolase family of enzymes in all living kingdoms, the sequence-based search for ochratoxin-hydrolyzing enzymes is limited by the abundance of sequence homologs in all the proteomes. In this work, we proposed the application of a structure-based mining protocol based on the generation of homology models to filter out the potential ochratoxin-degrading enzymes and to characterize their molecular features that could be related with their catalytic activities. Similar structural-based approaches have been recently applied for the screening of ochratoxin-binding domains and proteins in structural databases [59].

Our starting point was the work by Dobritzsch and coworkers, where the authors described the only atomic structure of a Zn-dependent amido-hydrolase able to degrade ochratoxin and isolated from *A. niger* [30]. Despite the complete molecular characterization of the enzyme octameric structure and their catalytic parameters, the authors did not provide detailed information about the features of the substrate-binding pocket. In this work, we performed a complete study by substrate blind docking to characterize the residues involved in ochratoxin recognition and binding in the *A. niger* enzyme, using molecular models of OTA and OTB. Our model suggests that the configuration of the catalytic pocket resembles the observed in bacterial arginine amido-hydrolases, where the substrate-binding is ensured by complementary histidine residues different from those involved in the coordination of the Zn-binuclear center [60]. In contrast to other amido-hydrolases of known structure, the catalytic pocket of ochratoxinase from *A. niger* is enriched in hydrophobic amino acids and contains two residues (His191 and Lys351) able to establish cation–pi interactions with ochratoxin [61]. Cation–pi interactions have been described as essential events in the structural stability of hydrolytic enzymes such as beta-lactamases [62], as well as in the specificity of the substrate-binding and recognition by diverse metabolizing enzymes [63]. Our data suggested the importance of hydrophobic and cation–pi interactions in the stabilization of enzyme–substrate complexes, being an essential element in the overall configuration of the *A. niger* enzyme.

Additionally, the study of the short-range behavior of the *A. niger* ochratoxinase catalytic center by molecular dynamics allowed us to determine the substrate-binding characteristics of the enzyme. Our results are compatible with the existence of two different regions in the catalytic pocket of the enzyme that contributes to the stabilization of the enzyme–substrate complex and further hydrolysis (Figure 3). The residues involved in the stabilization of the enzyme–substrate complex are in the boundaries of the substrate-binding pocket and constituted by hydrophobic amino acids involved in hydrophobic and Van der Waals interactions with the substrate. The MM-PBSA method allowed us to determine that the catalytic amino acids have a negative impact on the stability of the enzyme–substrate complex, suggesting a fast catalytic cycle upon substrate binding. These results are also in agreement with the observation that in other metal-dependent amidohydrolases, the stabilization of the enzyme–substrate intermediate complexes is the rate-limiting step of the catalytic cycle [64].

Using a combined approach that joins sequence homology with the construction of atomic models, we selected a dataset of 83 different fungal proteins with ochratoxinase-like characteristics. Structure superposition and phylogenetic analysis of the dataset of fungal proteins pointed to the possibility of the existence of distinct molecular characteristics that could differentiate the ochratoxinase-like enzymes belonging to the proteomes of ochratoxin-producers from other fungal enzymes (Figure 4). At this point, we could not exclude the observation of a species effect where the enzymes produced by closely related organisms were clustered together, especially when considering that 95% of the ochratoxin-producing fungi belong to the *Aspergillus* or *Penicillium* genera [65]. However, further data extracted from substrate-docking analysis across all the protein models allowed us to dissect some specific features of this group of enzymes. Our data suggested that ochratoxinase-like enzymes from ochratoxin producers showed more favorable characteristics for substrate binding, demonstrated by a smaller catalytic pocket volume and increased stability of enzyme–substrate complexes (Figure 5). Amido-hydrolases from the ochratoxin-producing fungi also have a catalytic pocket where the hydrophobic-enzyme–substrate interactions are enhanced by the presence of positively charged amino acids that will contribute to the substrate recognition and binding. Similar substrate-binding pockets are also observed in a family of fungal dihydro-piriminidases, a group of cyclic amino-hydrolases where the enzyme–substrate interactions are supported by cation–pi interactions [66]. In this family of enzymes, the conservation of the substrate-binding pocket has been proposed to be responsible for the enzymatic specificity and family structure [67]. Interestingly, the study of surface electrostatics across the selected proteins did not show relevant differences between the enzymes from ochratoxin producers and non-producers at the catalytic center (Figure 5). In contrast to amido-hydrolases involved in the degradation of polymeric substrates such as polyketides, the substrate-binding pocket of the studied ochratoxinase-like enzymes is located in a deep cavity flanked by a negatively charged surface patch [68].

Substrate recognition by enzymes is often driven by adaptable protein segments that can accommodate different chemical compounds [69]. This enzymatic promiscuity is especially relevant in the case of amido-hydrolases which share a very conserved protein skeleton that can catalyze hydrolysis of a wide range of substrates [70,71]. We studied the flexibility of the selected set of ochratoxinase-like enzymes by applying a normal mode analysis. This approach is based on a coarse-grained model and compared with classical molecular dynamics methods is less computer intensive [56]. The differential normal mode analysis results also demonstrated the clear differences in ochratoxinase-like enzymes from toxin producers and non-producers (Figure 6). These differences are clearly observable in the N-terminal region but also in a central protein segment that forms an alpha-helical lid over the catalytic pocket of the enzyme. The flexibility of the helical lid covering the active center quantified as the C-alpha fluctuation is increased in the enzymes from ochratoxin-producing fungi. This fact will help in the recognition and accommodation of organic substrates with aromatic rings as ochratoxins.

In summary, our data suggested that ochratoxin-producing fungi are a potential source of ochratoxin-degrading enzymes that harbor enhanced catalytic properties to act over the mycotoxin, demonstrated by the configuration and properties of the catalytic center and the flexibility of the surrounding residues to accommodate the substrate. These enzymes are interesting starting points for the development of new enzyme-based bioremediation strategies.

## Data Availability

Coordinates of the homology models are available as Supplementary Materials attached to the manuscript.

## Supplementary Materials

The following are available online at https://www.mdpi.com/article/10.3390/biom11071040/s1, Figure S1: Proposed mechanism for the hydrolysis of OTA by ochratoxinase-like enzymes with amido-hydrolase activity and harboring a binuclear Zn^2+^ center; Figure S2: Root mean square analysis of the molecular dynamics simulations trajectories comparing the ochratoxinase from *A. niger* in the apo-form with the enzyme–substrate complex; Figure S3: Radius of gyration, secondary structure and correlation analysis of the molecular dynamics results comparing the ochratoxinase from *A. niger* in the apo-form with the enzyme–substrate complex; Figure S4: Sequence alignment logo based on the structural features of the ochratoxinase-like enzymes selected from ochratoxin-producing fungi; Figure S5: Comparison of the ochratoxinase-like enzymes based on the individual analysis of the electrostatic surfaces in the atomic models; Table S1: Phyre2 homology modeling statistics and results; Table S2: Blind-docking results by CB-Dock; Table S3: Energy contribution of each amino acid in *A. niger* ochratoxinase to the substrate-binding as calculated by applying the MM-PBSA method to the molecular dynamics trajectories; Supplementary File ‘Refined_homology_atomic_models.zip’: ZIP-compressed file containing the atomic coordinates of all the refined homology models used in the study.
