# Supplementary material for: Systematic Structure-Based Search for Ochratoxin-Degrading Enzymes in Proteomes from Filamentous Fungi
Source: Biomolecules. 2021 Jul 16;11(7):1040. doi: 10.3390/biom11071040 (PMC8301969; doi:10.3390/biom11071040)
Supplement: Supplementary file 1 [file biomolecules-11-01040-s001.zip › Figure S1.pdf]

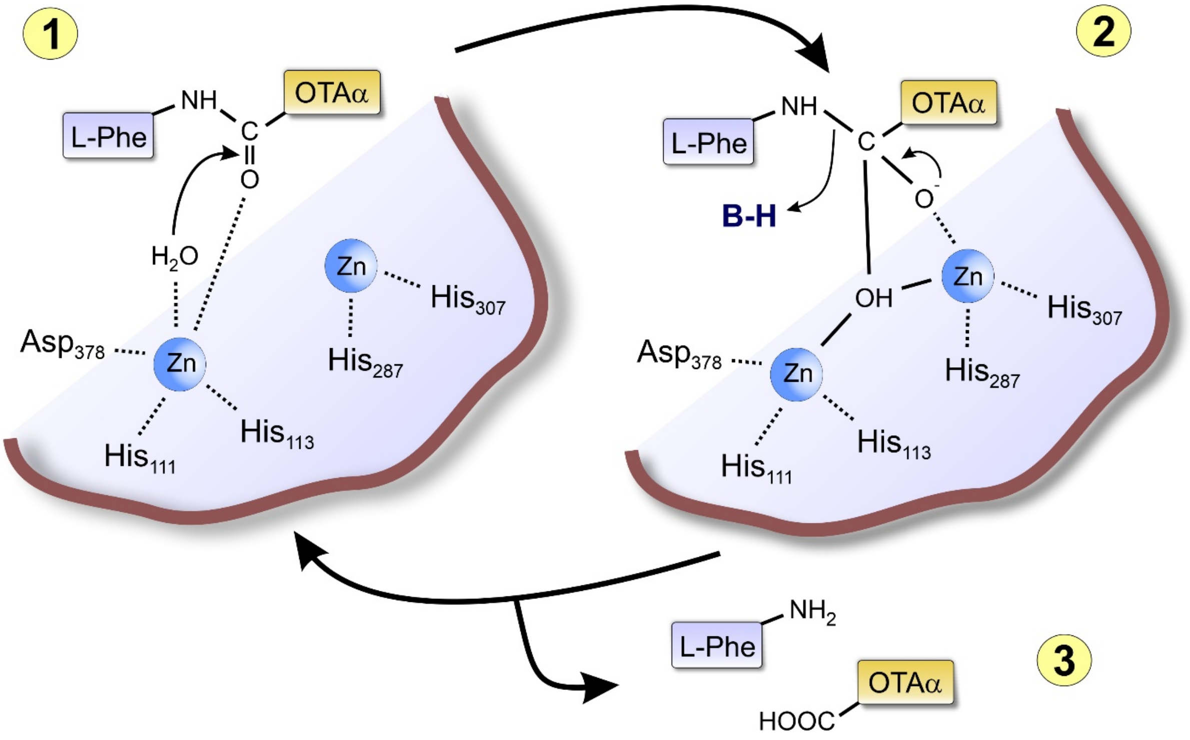

Figure S1. Proposed mechanism for the hydrolysis of ochratoxin A by ochratoxinase-like enzymes with amido-hydrolase activity and harboring a binuclear Zn^{2+} center. A water molecule that occupies one coordination site of a Zn^{2+} atom is activated by the presence of a coordinating aspartate residue of the same metal atom. The activated water molecule is responsible for a nucleophilic attack to the amide bond. The oxidized intermediate is stabilized by the presence of the second Zn^{2+} atom. Finally, the electrons of the amide bond are transferred to an electron acceptor, and the enzyme releases ochratoxin a and phenyl-alanine.
