# Supplementary material for: Systematic Structure-Based Search for Ochratoxin-Degrading Enzymes in Proteomes from Filamentous Fungi
Source: Biomolecules. 2021 Jul 16;11(7):1040. doi: 10.3390/biom11071040 (PMC8301969; doi:10.3390/biom11071040)
Supplement: Supplementary file 1 [file biomolecules-11-01040-s001.zip › Figure S2.pdf]

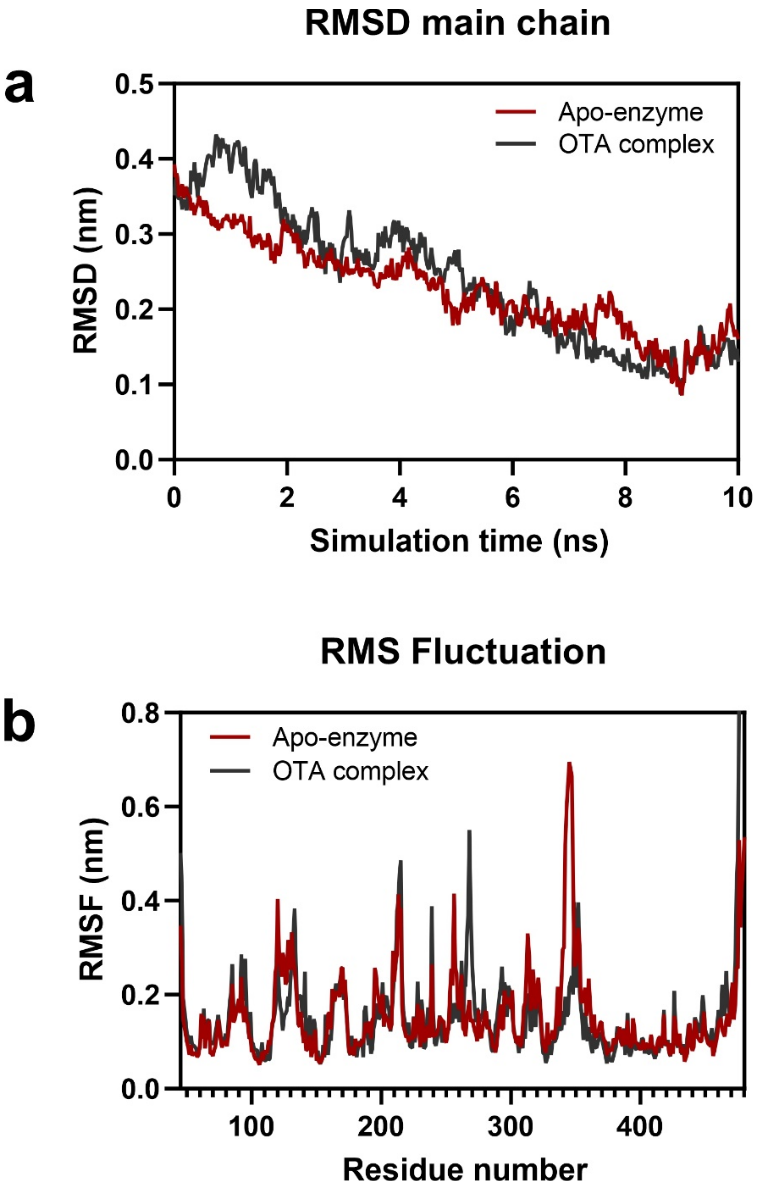

Figure S2. Root mean square analysis of the molecular dynamics simulation trajectories. (a) Root mean square deviation of the spatial positions of the main chain protein atoms in the ochratoxinase apoenzyme from *Aspergillus niger* (PDB code: 4C5Y) and the corresponding OTA-bound complex; (b) Average root mean square fluctuations of atomic positions from all the protein residues along the simulation in the same ochratoxinase apo-enzyme compared with the OTA complex.
