# Supplementary material for: Systematic Structure-Based Search for Ochratoxin-Degrading Enzymes in Proteomes from Filamentous Fungi
Source: Biomolecules. 2021 Jul 16;11(7):1040. doi: 10.3390/biom11071040 (PMC8301969; doi:10.3390/biom11071040)
Supplement: Supplementary file 1 [file biomolecules-11-01040-s001.zip › Figure S3.pdf]

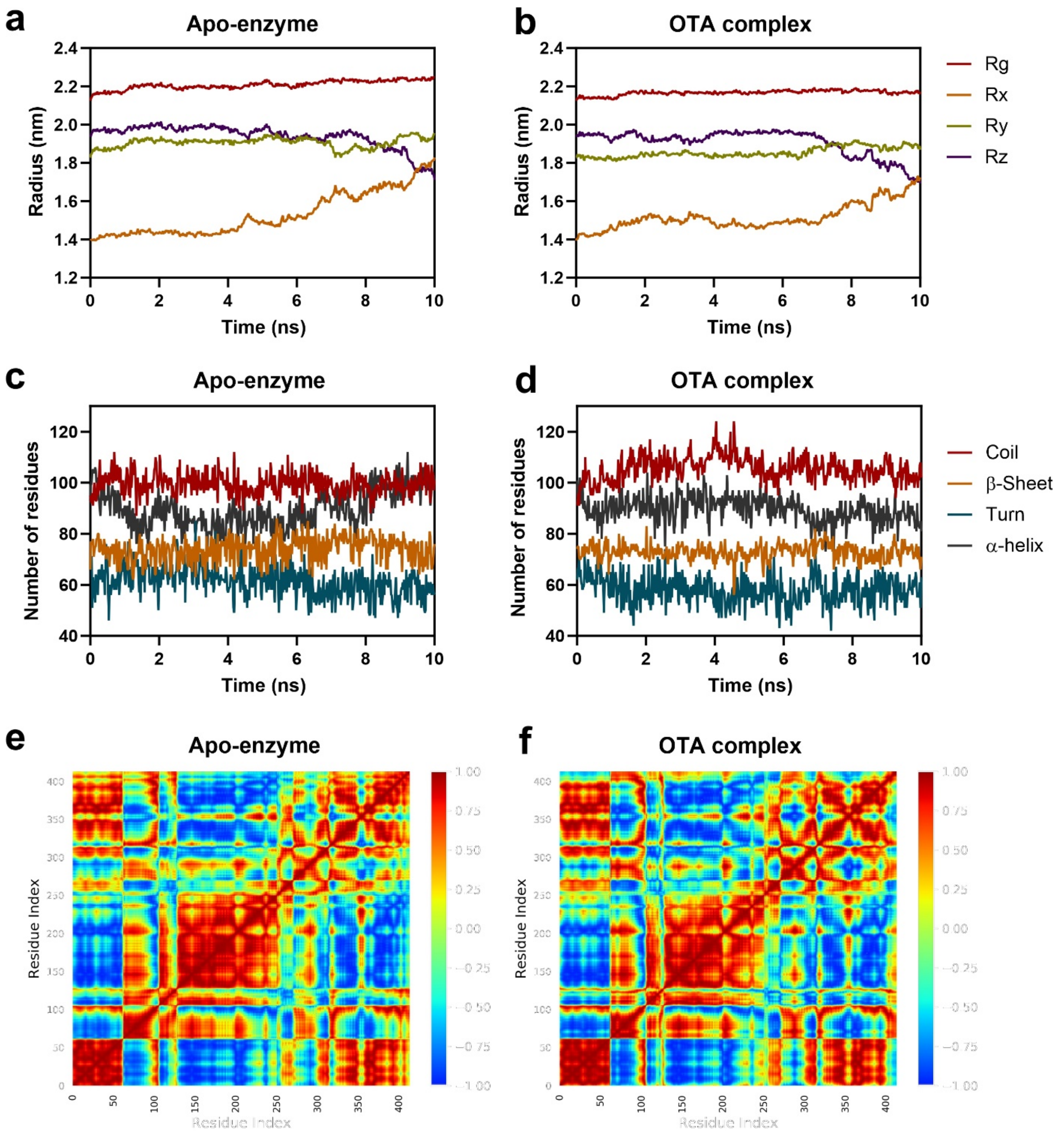

Figure S3. Radius of gyration, secondary structure, and correlation analysis of the molecular dynamics simulation results comparing the ochratoxinase from *Aspergillus niger* in the apo-form (PDB code: 4C5Y) and complexed with the ochratoxin A (OTA) substrate. (a, b) evolution of the radius of gyration showing the average protein radius (Rg) and the radius in the 3 cartesian axes (Rx, Ry and Rz) in the ochratoxinase apoenzyme and the corresponding OTA complex; (c, d) Fluctuations of the number of atoms included in different secondary structure elements (coil, beta-sheet, turn and alpha-helix) in the ochratoxinase apo-enzyme and OTA complex; (e, f) average correlation of the C-alpha atoms coordinates during the simulation in the apo-enzyme and OTA complex.
