# Supplementary material for: Systematic Structure-Based Search for Ochratoxin-Degrading Enzymes in Proteomes from Filamentous Fungi
Source: Biomolecules. 2021 Jul 16;11(7):1040. doi: 10.3390/biom11071040 (PMC8301969; doi:10.3390/biom11071040)
Supplement: Supplementary file 1 [file biomolecules-11-01040-s001.zip › Figure S4.pdf]

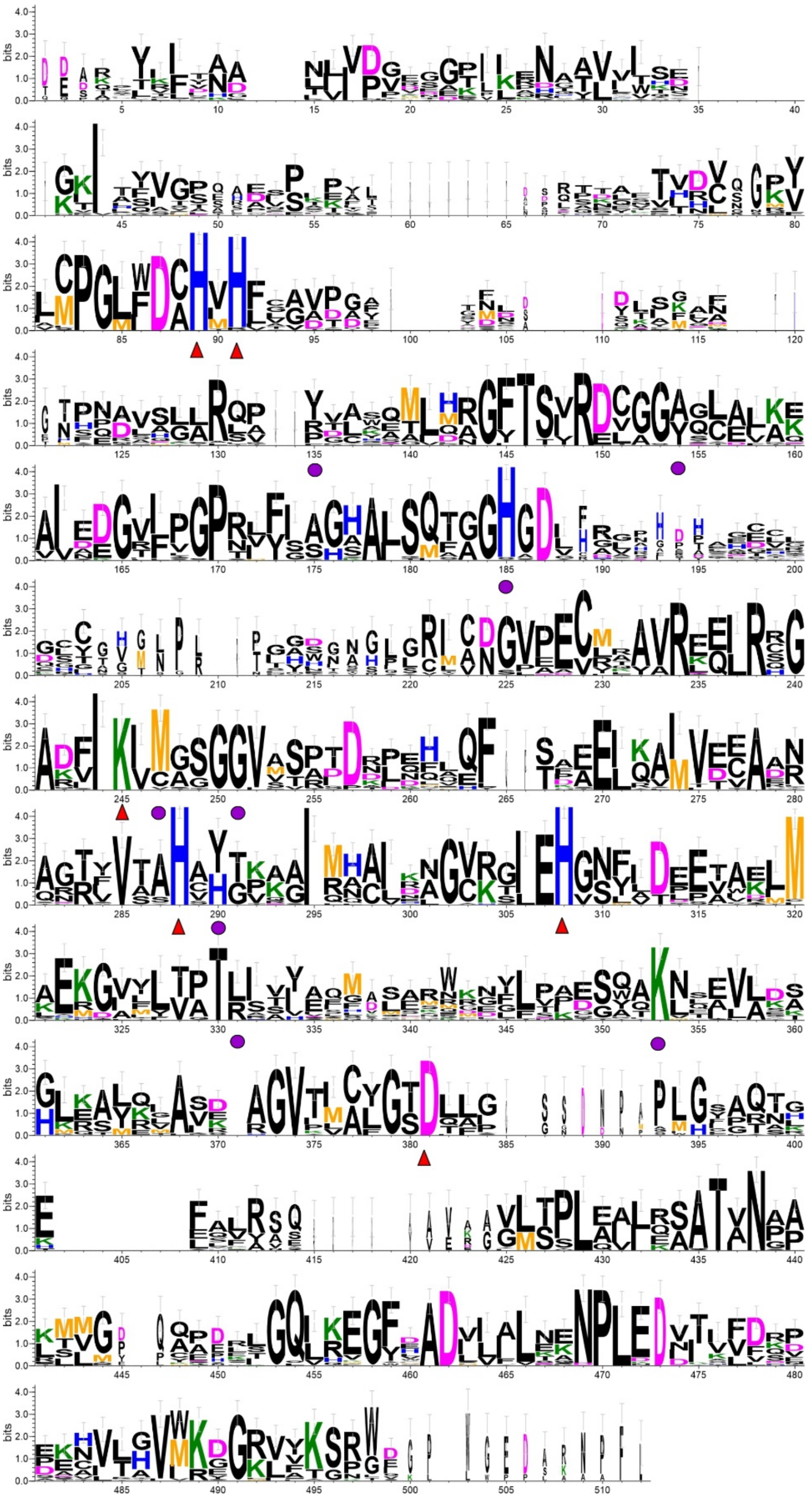

Figure S4. Sequence alignment logo based on the structural features of the ochratoxinase-like enzymes selected from ochratoxin-producing fungi. Residues involved in the coordination of catalytic Zn^{2+} atoms are labelled with red triangles, whereas the residues involved in the structure of the substrate-binding pocket and putative interactions with ochratoxins are labelled by magenta circles.
