# Supplementary material for: Systematic Structure-Based Search for Ochratoxin-Degrading Enzymes in Proteomes from Filamentous Fungi
Source: Biomolecules. 2021 Jul 16;11(7):1040. doi: 10.3390/biom11071040 (PMC8301969; doi:10.3390/biom11071040)
Supplement: Supplementary file 1 [file biomolecules-11-01040-s001.zip › Figure S5.pdf]

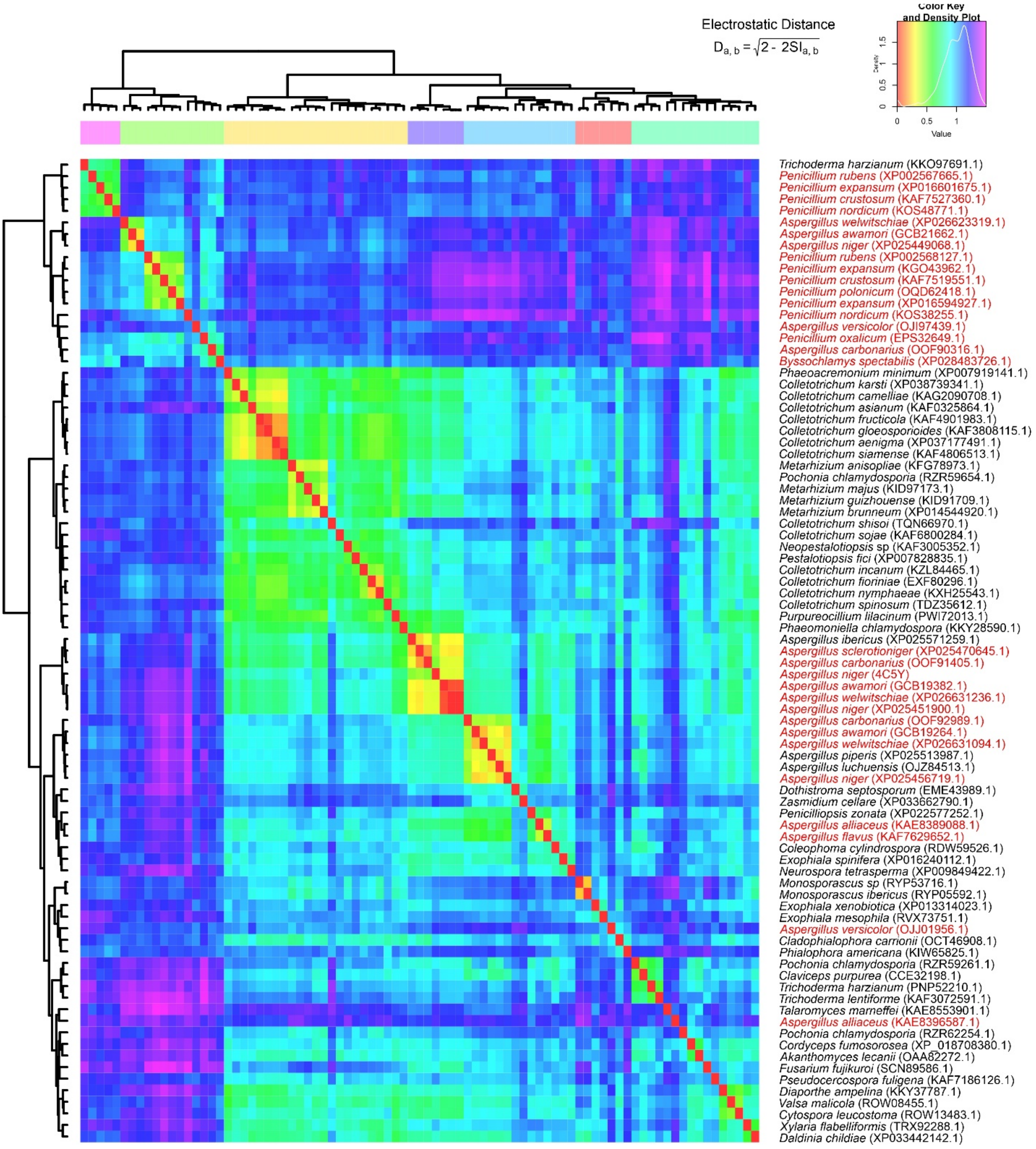


**Figure S5. Comparison of the ochratoxinase-like enzymes based on the individual analysis of electrostatic surfaces in the atomic models.** Hierarchical clustering analysis of the electrostatic distance matrix for the selected group of carboxylic ester hydrolases structures was calculated by the webPIPSA algorithm and the Poisson-Boltzman equation. The enzymes are identified by their producing fungal strain, where the ochratoxin-producing species are depicted in red color.
